# Effects of an eight-week mindfulness-informed psychoeducational intervention delivered during physical education lessons on self-reported aggression and impulsivity in adolescents

**DOI:** 10.3389/fpsyg.2026.1958309

**Published:** 2026-10-12

**Authors:** Walid Selmi, Achraf Hammami, Said Ben Hassen, Yassine Negra, Raouf Hammami, Roland van den Tillaar

**Affiliations:** 1 Higher Institute of Sport and Physical Education of Ksar-Said, University of Manouba, Manouba, Tunisia; 2 Research Laboratory (LR23JS01) Sport Performance, Health & Society, Higher Institute of Sport and Physical Education of Ksar-Said, University of Manouba, Manouba, Tunisia; 3 Tunisian Research Laboratory ‘Sports Performance Optimization' (CNMSS-LR09SEP01), National Center of Medicine and Science in Sports (CNMSS), Tunis, Tunisia; 4 Department of Sport Sciences and Physical Education, Nord University, Levanger, Norway

**Keywords:** behavioral regulation, coping strategies, school-based intervention, self-control, stress management, well-being

## Abstract

Aggression and impulsivity are prevalent behavioral tendencies in adolescents that may negatively affect academic performance, social interactions, and engagement during physical education (PE). Although mindfulness-based interventions have shown promise for improving emotional and behavioral regulation, evidence of their effectiveness when delivered within school PE settings remains limited. This study investigated the effects of an 8–week mindfulness-informed psychoeducational intervention delivered during PE lessons on self–reported aggression and impulsivity among high school students. Thirty-nine students (16.05 ± 0.55 years; 51% boys) were randomly assigned to either a mindfulness group (n = 20) or a control group (n = 19). The mindfulness group participated in two brief sessions per week incorporating mindfulness-oriented practices, including focused attention, conscious breathing, and body awareness, together with psychoeducational and sport-related psychological strategies, including positive affirmations, self-encouragement, and performance imagery, whereas the control group followed the standard PE curriculum. General self-reported aggression and impulsivity were assessed before and after the intervention using the Aggression Questionnaire (AQ) and the Barratt Impulsivity Scale (BIS-11). Data were analyzed using repeated-measures analysis of variance and effect sizes. Significant group–by–time interactions were observed for verbal aggression, physical aggression, hostility, non-planned impulsivity, and total impulsivity, indicating that changes over time differed between the mindfulness and control groups for these outcomes. The direction of these interactions was characterized by reductions in verbal aggression (-33.5%), physical aggression (-32.4%), non-planned impulsivity (-13.1%), and total impulsivity (-10.5%) in the mindfulness group, while the control group showed increases in verbal aggression and hostility and no significant improvements in impulsivity. Although motor impulsivity decreased by 11.6% within the mindfulness group, the group–by–time interaction. was not significant; therefore, this within-group change was not interpreted as evidence of a differential intervention effect. These findings suggest that a brief mindfulness-informed psychoeducational intervention delivered during PE lessons was associated with differential changes in verbal aggression, physical aggression, hostility, non-planned impulsivity, and total impulsivity relative to the control condition. These findings support the potential feasibility of integrating mindfulness-informed psychoeducational strategies within school PE while recognizing that the outcomes assessed general dispositions rather than behaviors occurring specifically during PE lessons.

## 1 Introduction

Aggressiveness and impulsivity are common behavioral traits in school settings that can negatively affect academic performance and interpersonal relationships (Carlo et al., 2011). Aggressive behavior typically involves physical or verbal hostility, whereas impulsivity refers to acting without sufficient forethought, often leading to conflicts and poor decision-making (Moeller et al., 2001). These behaviors are particularly prominent during adolescence, a developmental stage characterized by increased emotional reactivity and still-maturing self-regulation abilities.

Impulsivity and aggressiveness arise from interacting biological, psychological, and environmental factors. Deficits in executive functioning and disorders such as ADHD are associated with poor emotional and behavioral control (Connor, 2012). Environmental influences, including family conflict, inconsistent parenting, exposure to violence, peer rejection, and aggressive peer groups, may further contribute to maladaptive behaviors (Bandura and Walters, 1977; Dishion et al., 1996; Loeber and Stouthamer-Loeber, 1998). Additionally, chronic stress linked to lower socioeconomic status may exacerbate emotional dysregulation and impulsive responses (Bradley and Corwyn, 2002).

Physical education may represent a particularly relevant setting for interventions targeting emotional and behavioral regulation, as competition, social interaction, and performance demands can elicit emotional and behavioral responses. These behaviors are often intensified during physical education sessions, where competition and performance demands may increase emotional and behavioral reactions. Adolescents with higher aggressiveness tend to rely on maladaptive coping strategies, including hostility and impulsive reactions, particularly in competitive sports contexts (Zhu et al., 2022). The emotionally dynamic nature of physical education activities may further reinforce impulsive behaviors among students with poor self-regulation (Guerra et al., 2011). Consequently, interventions targeting emotional regulation in physical education settings may help improve behavioral control and classroom climate (Rico-Gonzalez, 2023).

School-based interventions such as social-emotional learning programs have shown positive effects on emotional regulation, empathy, and aggressive behavior reduction (Durlak et al., 2011). Among these approaches, mindfulness-based interventions have gained attention for improving emotional regulation and reducing anxiety, anger, and impulsive behaviors (Fix and Fix, 2013; Amutio et al., 2014; Zenner et al., 2014). (Franco et al. 2016) reported reductions in impulsivity and aggression among adolescents following mindfulness training. However, interventions incorporating mindfulness-oriented practices may also include complementary psychoeducational or psychological strategies, such as self-compassion, positive self-talk, and imagery. Such multicomponent approaches should be distinguished from interventions consisting exclusively of mindfulness practice, because the contribution of individual components to behavioral outcomes cannot be readily isolated.

However, despite these promising findings, controlled studies examining mindfulness-informed psychoeducational interventions delivered within physical education settings and their effects on adolescents' general aggression and impulsivity remain scarce. Furthermore, although improvements in present-moment awareness, attentional control, and emotional regulation represent plausible mechanisms through which mindfulness-oriented practices may influence aggression and impulsivity, these mechanisms require direct assessment to be established empirically. Therefore, the present study aimed to evaluate the effects of an 8-week mindfulness-informed psychoeducational intervention delivered during physical education sessions on adolescents' general self-reported aggression and impulsivity. The intervention combined mindfulness-oriented practices, such as focused attention, conscious breathing, and body awareness, with complementary psychoeducational and sport-related psychological strategies, including positive affirmations, self-encouragement, and performance imagery. Based on previous findings (Franco et al., 2016; Hamidi et al., 2020; O'dean et al., 2025), it was hypothesized that students participating in the mindfulness-informed psychoeducational intervention would demonstrate greater reductions in self-reported impulsivity and aggressiveness than students in the control condition. This study is situated within the fields of sport psychology, adolescent behavioral regulation, school-based psychological interventions, and physical education. Specifically, it examines the potential effects of a mindfulness-informed psychoeducational intervention integrated into physical education on adolescents' psychological and behavioral outcomes, including aggression and impulsivity. By incorporating brief mindfulness-informed activities within the school physical education context, the study contributes to the understanding of how psychologically informed strategies can be implemented in educational and sport-related settings to support adolescents' behavioral regulation and psychosocial well-being.

## 2 Materials and methods

### 2.1 Participants

A total of thirty-nine students (Age = 16.05 ± 0.55 years; Height = 170.84 ± 8.68 cm; Body Mass = 62.15 ± 8.61 kg;) from the same class at a secondary high school in downtown Tunis participated in this study. All 39 students assessed for eligibility met the inclusion criteria and agreed to participate; therefore, no participants were excluded before randomization. Because sex-stratified randomization was used, the mindfulness group included 13 boys (65%) and 7 girls (35%), while the control group included 12 boys (63.2%) and 7 girls (36.8%). Standardized differences were calculated as the absolute difference in group means divided by the pooled standard deviation for continuous variables, whereas for categorical variables they were calculated using the appropriate standardized proportion difference. A standardized difference of < 0.10 was considered indicative of good balance between groups, with larger values indicating greater between-group imbalance. Participants were randomly assigned to either the mindfulness group (*n* = 20) or control group (*n* = 19), using a sex-stratified randomization procedure to ensure a balanced distribution of boys and girls between groups. Separate randomization sequences were generated for boys and girls using a computer-generated random sequence in Microsoft Excel, with participants allocated in a 1:1 ratio whenever possible. The randomization sequence was generated by a researcher who was not involved in the assessments, while participant enrollment was performed by the principal investigator. Group allocation was implemented by the researcher who generated the randomization sequence. No formal allocation concealment procedure was used. Twenty participants were allocated to the mindfulness-informed psychoeducational intervention group and 19 to the control group (Figure 1). Biological maturity was estimated for each participant using the maturity offset method (Moore et al., 2015), which estimates years from peak height velocity. Biological maturity was assessed to characterize the developmental status of the sample and to evaluate potential baseline differences between groups. Group-specific baseline characteristics, including age, sex, maturity offset, height, and body mass, together with standardized differences between groups, are presented in Table 1.

**Figure 1:**
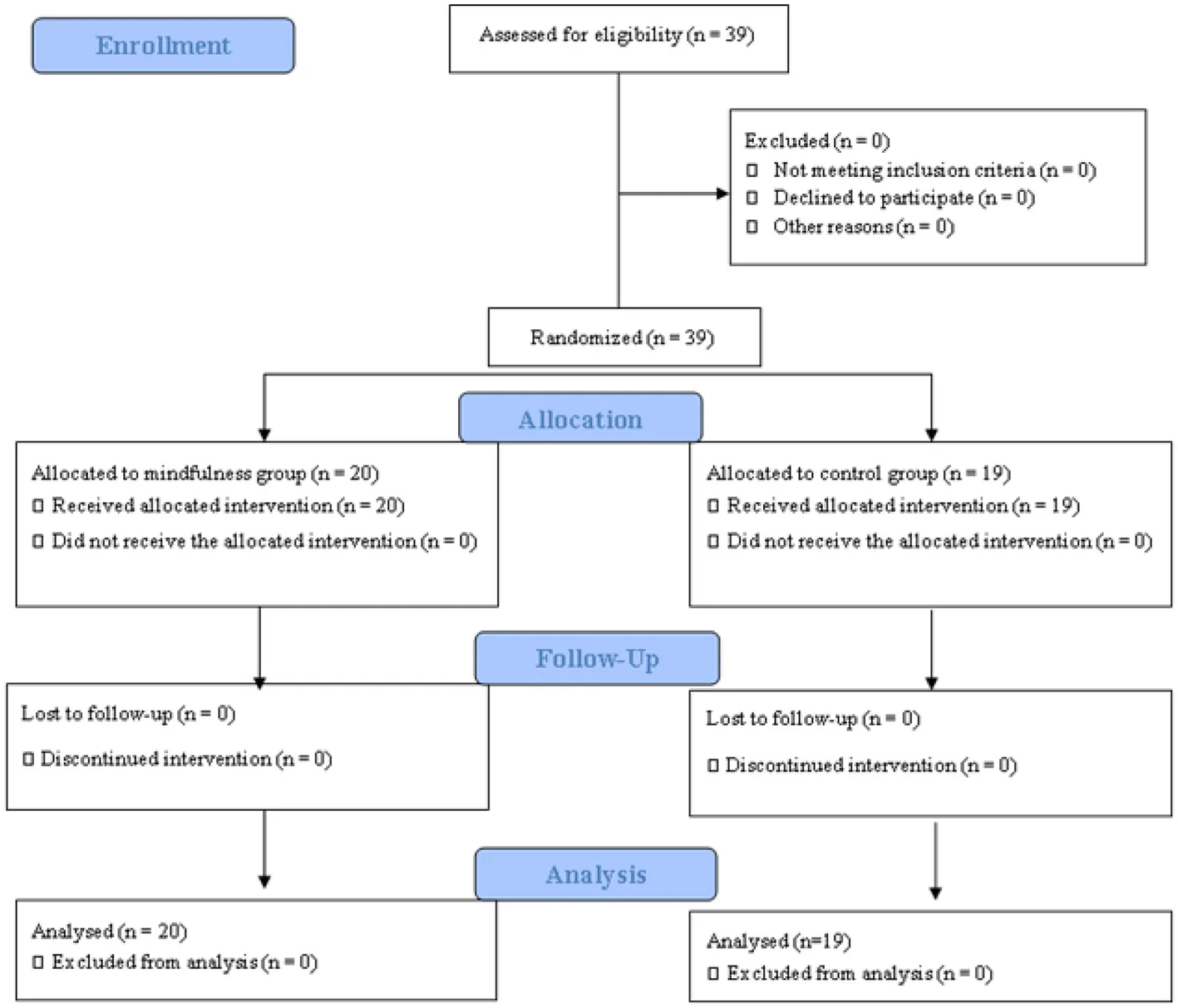
The consolidated standards of reporting trials flow diagram with detailed information on the participant flow.

**Table 1:** Mean ± SD baseline characteristics of participants by group.

Variables	Mindfulness group (*n* = 20)	Control group (*n* = 19)	Standardized difference
Age (years)	16.10 ± 0.55	16.00 ± 0.58	0.18
Body mass (kg)	63.75 ± 8.84	60.47 ± 8.27	0.38
Height (cm)	172.00 ± 8.27	169.63 ± 9.17	0.27
Maturity offset (years)	2.00 ± 0.46	1.81 ± 0.76	0.30
BMI (Kg/m^2^)	21.55 ± 0.12	21.02 ± 0.09	0.25
Age at peak height velocity (years)	14.10 ± 0.61	14.19 ± 0.49	−0.15

Values are presented as mean ± standard deviation (SD). BMI, body mass index (kg/m^2^); maturity offset, years from peak height velocity (PHV); age at peak height velocity, estimated age at PHV. Standardized difference represents the standardized mean difference between the mindfulness and control groups, with positive values indicating higher values in the mindfulness group and negative values indicating higher values in the control group.

Pre-test measurements were obtained for all participants before the intervention. These included self-reported assessments of impulsivity and aggressiveness using standardized questionnaires: the Barratt Impulsivity Scale (BIS-11) and the Aggression Questionnaire (AQ).

All participants and their legal guardians were fully informed about the study objectives, procedures, potential benefits, and risks. Written informed consent was obtained from each participant's parent or legal guardian, and written assent was obtained from all participating adolescents before study enrollment. The study was approved on Monday 12 February 2026 by the Local Ethics Committee of the National Center of Medicine and Science of Sports of Tunis, Tunisia (CNMSS-LR09SEP01-N°O110/2026) and conducted in accordance with the Declaration of Helsinki. None of the participants or their parents/legal guardians reported a history of diagnosed psychological, musculoskeletal, neurological, or orthopedic disorders during the 6 months preceding the study or during the intervention period. This information was obtained through self-report and parental/legal guardian report during the eligibility assessment and was not established through formal clinical or psychiatric assessment. Accordingly, the criterion referred to the absence of self-reported or parent-reported diagnosed disorders, rather than the clinical absence of psychological symptoms or disorders. The study was not prospectively registered. A study protocol was prepared before the intervention but was not prospectively registered or published.

### 2.2 Procedures

Prior to data collection, participants were informed about the objectives of the study and the voluntary nature of their participation. Pre-test assessments were administered 1 week before the intervention period. Participants completed the Arabic versions of the Aggression Questionnaire and the Barratt Impulsivity Scale individually in a quiet and supervised classroom environment to minimize distractions and ensure standardized administration conditions. Participants completed both questionnaires according to the standard instructions of the respective versions administered and were not specifically instructed to respond with reference to behaviors occurring during physical education lessons. No PE-specific reference period was imposed, and participants responded according to the standard instructions of the respective questionnaires. Accordingly, the AQ and BIS-11 were interpreted as measures of general self-reported aggression and impulsivity rather than behaviors occurring within a specific PE lesson or PE-related time period.

Following the baseline assessment, participants were assigned to either the mindfulness intervention group or the control group. Given the nature of the intervention, participants and the facilitator delivering the mindfulness sessions could not be blinded to group allocation. Questionnaire administrators were not blinded to group allocation. The data analyst was not blinded to group allocation, and PE teachers were aware of group allocation.

The mindfulness group participated in an 8-week psychoeducational mindfulness program integrated into regular physical education classes and conducted twice weekly. Each session lasted approximately 5–15 min and incorporated several mindfulness-based techniques, including focused attention exercises, breathing synchronization, body awareness activities, and self-compassion practices, following the recommendations of Birrer and Morgan (2010). The intervention activities were designed to promote emotional awareness, attentional focus, self-control, and adaptive coping strategies during physical activity and competitive situations.

Attendance and participation were monitored throughout the intervention period. All mindfulness exercises were delivered by a trained facilitator to ensure consistency and adherence to the intervention protocol. The control group continued following the standard physical education curriculum without exposure to mindfulness activities.

Post-test assessments were conducted during the week immediately following completion of the 8-week intervention using the same instruments and procedures employed during the pre-test phase to ensure methodological consistency. No adverse events or injuries related to the intervention were reported during the study period.

### 2.3 Measures

#### 2.3.1 Aggression Questionnaire

The Aggression Questionnaire (AQ) (Buss and Perry, 1992), Arabic version adapted by Fekih-Romdhane et al. (2023), was used to assess aggressiveness. The Arabic version was not specifically validated among Tunisian adolescents; therefore, the use of this instrument in the present population should be considered when interpreting the findings. No additional cultural or linguistic adaptation was performed for the present study, as the previously published Arabic version was administered without modification. The AQ includes 29 items rated on a five-point Likert scale ranging from 1 (very few times) to 5 (lots of times), distributed across four dimensions: Physical Aggression (nine items), Verbal Aggressiveness (five items), Hostility (eight items), and Anger (seven items). The frequency-based response anchors reported here correspond exactly to those used in the Arabic version administered to participants. Although the original AQ commonly uses characteristic/uncharacteristic response anchors, no modification of the response format was made by the authors of the present study. Reverse-scored items were recoded before calculating the corresponding subscale and total scores, in accordance with the scoring procedure of the Arabic AQ version. Scores for each dimension were calculated by summing the responses to the items within the corresponding subscale, with higher scores indicating greater levels of aggressiveness in that domain. The internal consistency for the subscales ranged from α = 0.55 to 0.72.

#### 2.3.2 Barratt Impulsivity Scale

The Barratt Impulsivity Scale (BIS-11) (Patton et al., 1995) is a widely used instrument that measures impulsivity across three domains: Cognitive Impulsivity (eight items) – the tendency to make rapid decisions without careful consideration; Motor Impulsivity (10 items) – the tendency to act on the spur of the moment; and Non-planned Impulsivity (12 items) – reflecting lack of future-oriented planning. Items are scored on a four-point Likert scale (1 = rarely/never to 4 = almost always/always). The Tunisian adaptation reported by Ellouze et al. (2013) was used in the present study. Item scoring, reverse-scoring procedures, and subscale definitions followed exactly those of the validated Tunisian version, without modification. Reverse-scored items were recoded before calculating the corresponding subscale and total scores, in accordance with the scoring procedure of the Tunisian version. Subscale scores were summed to yield total impulsivity scores. The Tunisian version demonstrated acceptable internal reliability (Cronbach's α = 0.66–0.72).

### 2.4 Mindfulness-informed psychoeducational intervention

The mindfulness group participated in an 8-week mindfulness-informed psychoeducational intervention, delivered twice weekly during regular physical education (PE) sessions (Tables 2 and 3). Each intervention session lasted 5–15 minutes and included focused-attention exercises, breathing synchronization, body awareness, and self-compassion practices (Birrer and Morgan, 2010). The intervention also incorporated psychoeducational and sport-related psychological strategies, including positive affirmations, performance visualization, self-encouragement, and sport-success imagery. Accordingly, the intervention was conceptualized as a multicomponent mindfulness-informed psychoeducational program rather than as a purely mindfulness-based intervention.

**Table 2:** Mindfulness program: weekly objectives.

Weeks	Objectives	Description
Weeks 1–2	Orientation and awareness	•Introduction to mindfulness and the program •Awareness of breathing and bodily sensations. •Formal and informal mindfulness practice
Weeks 3–4	Mindfulness practice in the body	•Exploration of bodily sensations and movements •Mindful yoga to develop body awareness •Practice of mindful walking
Weeks 5–6	Mindfulness practice in thoughts and emotions	•Observing thoughts and emotions without judgment. •Exploration of mindful communication. •- Meditation techniques to cultivate kindness.
Weeks 7–8	Integration and planning for the future	•Reflection on changes and personal discoveries •Application of mindfulness in daily life •Planning to maintain a regular practice in the future

**Table 3:** Mindfulness program: weekly sessions and exercises.

Week & Theme	Sessions	Exercises	Duration
**Week 1** Introduction to Mindfulness	1: What is mindfulness?	Conscious breathing (slowly breathing while focusing on the breath).	10 min
	2: Connecting with the body	Quick body scan while sitting or standing.	10 min
**Week 2** Mindfulness of the body	3: Mindfulness in movement	Mindful walking (walking slowly while paying attention to the movements of the feet and legs).	10 to 15 min
	4: Mindfulness in stretching	Conscious stretching (stretching each part of the body while focusing on sensations).	15 min
**Week 3** Breathing and concentration	5: Deep breathing	Abdominal breathing (breathing deeply into the belly and observing the movement).	10 min
	6: Breathing and performance	Breathing synchronized with movement (linking breath with physical activity, such as jumping rope or running).	15 min
**Week 4** Managing thoughts	7: Observing thoughts	Sitting in silence, observing thoughts without judgment for a few minutes.	10 min
	8: Refocusing during sports	Mindful pause (taking a 1-2 minute mindful break during physical activity).	1 to 2 min/per exercise
**Week 5** Managing emotions	9: Recognizing emotions	Examining emotions after an exercise (observing and naming the emotions felt).	15 min
	10: Managing emotions during performance	Emotional check-in (taking a moment to ask oneself, “How do I feel?” during an activity).	10 min
**Week 6** Self-compassion	11: Self-compassion and performance	Self-compassion (repeating a kind phrase to oneself after a difficulty).	1 min/per exercise
	12: Encouraging oneself	Positive affirmations during physical activity.	1 min/per exercise
**Week 7** Visualization	13: Visualization and mindfulness	Visualizing a sports action before executing it (e.g., visualizing a perfect throw before performing it).	1 min/per exercise
	14: Visualizing success	Visualizing a moment of sports success and focusing on the associated bodily sensations.	2 min
**Week 8** Integration and Autonomy	15: Integrating mindfulness into sports	Brief summary of the practices learned and how to apply them in activities.	5 min
	16: Autonomy in practice	Independent session (students choose their preferred mindfulness practices and perform them alone).	5 to 10 min

The theoretical rationale was that focused attention, conscious breathing, and body awareness were intended to promote present-moment awareness and attentional control. Self-compassion and self-encouragement were intended to facilitate adaptive responses to negative thoughts and emotions, whereas positive affirmations were used to reinforce constructive self-directed cognitions. Performance visualization and sport-success imagery were incorporated to support attentional preparation, emotional regulation, and adaptive coping in PE and sport-related situations.

The intervention was designed to help students: (1) maintain awareness of their thoughts and emotions during physical activity; (2) regulate emotional responses to competitive or stressful situations; (3) improve attentional focus and self-control during PE tasks; and (4) develop positive coping strategies and self-encouragement techniques.

The standard PE curriculum consisted of [insert the actual PE activities taught during the study, e.g., specific team sports, individual sports, fitness/conditioning activities, technical skill development, and/or games]. These activities were delivered according to the school's regular PE curriculum and were conducted by the same PE teacher throughout the study.

The control group continued to participate in the standard PE curriculum throughout the 8-week study period and received no mindfulness-informed or other psychoeducational activities. Both groups attended two PE sessions per week, with each PE lesson lasting approximately 90 min. Thus, the control group received approximately 1,440 min of regular PE exposure over the 8-week intervention period.

For the intervention group, the 5–15-min mindfulness-informed psychoeducational activities replaced an equivalent portion of the regular 90-min PE lesson rather than being added to it. Consequently, the intervention group received approximately 1,200–1,360 min of standard PE exposure, depending on whether sessions lasted 15 or 5 min, in addition to 80–240 min of mindfulness-informed psychoeducational activities across the 16 sessions. The control group therefore received approximately 1,440 min of standard PE exposure, whereas the intervention group received the same total scheduled PE-session time but with a portion allocated to the mindfulness-informed intervention.

The facilitator who delivered the mindfulness-informed intervention was also the students' regular PE teacher. The facilitator was a qualified physical education teacher with experience in delivering mindfulness-based exercises and had received specific training in mindfulness techniques, including focused attention, breathing exercises, body awareness, and self-compassion practices. The facilitator was aware of the study hypotheses. To support intervention consistency, all sessions followed a predefined structure and standardized intervention content, and the same facilitator delivered all mindfulness-informed sessions throughout the 8-week intervention.

Pre-test measurements were conducted 1 week before the intervention. Post-test assessments were carried out during the week following completion of the 8-week program, using the same instruments and procedures as the pre-test. Both groups completed the questionnaires individually in a quiet, supervised classroom setting to minimize distractions.

Attendance and participation were recorded for all intervention sessions. The intervention comprised 16 planned sessions (two sessions per week for 8 weeks), and attendance was recorded at each session. Participant adherence was calculated as the number of sessions attended divided by the 16 scheduled sessions × 100. All 16 planned sessions were delivered as intended. Participants attended a mean of 16 sessions (range: 16–16), corresponding to an attendance rate of 100%. Adequate adherence was defined as attendance at ≥80% of scheduled sessions.

No formal quantitative fidelity checklist or independent fidelity assessment was used. Intervention fidelity was supported through standardized content, a predefined session structure, and delivery by the same trained facilitator. Specifically, no session-by-session fidelity checklist, independent observation, audio/video recording, or independent fidelity rating was used.

Participants were not instructed to practice mindfulness outside the scheduled intervention sessions, and independent mindfulness practice was not monitored. Participant engagement beyond session attendance was not formally assessed using a standardized measure. Potential adverse psychological reactions were not systematically assessed using a standardized instrument; however, no participant spontaneously reported psychological discomfort or requested discontinuation of the intervention. No adverse events or injuries related to the intervention were reported.

### 2.5 Statistical analyses

Data are presented as means and standard deviations (SD). Normality assumption was tested using the Shapiro-Wilk test and confirmed (*p* > 0.05). Baseline characteristics, including age, sex, maturity offset, height, body mass, and BMI, were summarized separately for each group. Standardized differences were calculated to quantify the magnitude of potential baseline imbalance between groups rather than relying solely on non-significant baseline hypothesis tests.

To establish the effect of the interventions on the dependent variables, a 2 (group: mindfulness and control group) × 2 (time: pre, post) ANOVA with repeated measures was determined for each parameter. When group × time interactions reached the level of significance (i.e., significant F value), Holm-Bonferroni post-hoc tests were used. The alpha level of significance was set at *p* < 0.05. Given the small sample size, maturity offset and sex were not included as additional covariates or interaction terms in the primary analyses to avoid overparameterization. The study was not specifically powered to examine sex- or maturity-specific intervention effects; therefore, no subgroup analyses according to sex or maturity were performed. Effect sizes for ANOVA were reported as partial eta squared (η_*p*_^2^) where 0.01 < $\eta_{p}^{2}$ < 0.06 constitutes a small effect, a medium effect when 0.06 < $\eta_{p}^{2}$ < 0.14 and a large effect when $\eta_{p}^{2}$ > 0.14 (Cohen, 1988). When comparing pairwise post hoc, the magnitude of the training effect, Cohen's d was used as effect size. According to Hopkins et al. (2009), Cohen's d values were interpreted as trivial (< 0.2), small (0.2–0.6), moderate (0.6–1.2), large (1.2–2.0), very large (2.0–4.0), and extremely large (>4.0). No a priori sample-size calculation was conducted. Therefore, a sensitivity analysis was performed based on the final sample of 39 participants (mindfulness group, *n* = 20; control group, *n* = 19). Assuming an alpha level of 0.05, 80% statistical power, a correlation of 0.50 between repeated measures, and a no sphericity correction of 1, the analysis indicated that the study was able to detect a minimum group × time interaction effect of Cohen's *f* = 0.23. All data analyses were performed using JASP (version 0.97.1, University of Amsterdam).

## 3 Results

All participants received the treatment as allocated. All 16 scheduled mindfulness sessions were delivered as planned. Participants in the mindfulness group attended a mean of 16 sessions (range: 16–16), with no missed sessions, corresponding to an attendance rate of 100%. Thus, all participants met the predefined criterion for adequate adherence. No test-related injuries were reported. No intervention-related adverse events were reported; however, adverse psychological reactions were not systematically assessed using a standardized instrument. At baseline, no significant between-group differences were observed in the anthropometric characteristics. Similarly, no between-group differences were recorded at baseline for any measure of psychological tests (*p* > 0.05).

### 3.1 Aggression Questionnaire

Significant group-by-time interaction effects were observed for verbal aggression (*F*_(1, 37)_ = 40.7, *p* < 0.001, $\eta_{p}^{2}$ = 0.52), physical aggression (*F*_(1, 37)_ = 48.1, *p* < 0.001, $\eta_{p}^{2}$ = 0.57), and hostility (*F*_(1, 37)_ = 4.68, *p* = 0.037, $\eta_{p}^{2}$ = 0.11), indicating that changes over time differed between the mindfulness and control groups for these outcomes. A significant main effect of time was also observed for physical aggression (*F*_(1, 37)_ = 12.8, *p* < 0.001, $\eta_{p}^{2}$ = 0.26), and a significant main effect of group was found for verbal aggression (*F* = 4.56, *p* = 0.039, $\eta_{p}^{2}$ = 0.11). Post hoc comparisons were used to characterize the direction of the significant interactions. In the mindfulness group, verbal aggression decreased by 33.5% (Cohen's *d* = 1.05) and physical aggression by 32.4% from pre- to post-test. In contrast, verbal aggression increased by 12.3% (Cohen's *d* = 0.56) in the control group (Figure 2). For hostility, the significant group-by-time interaction reflected different patterns of change between groups, with hostility scores decreasing in the mindfulness group and increasing in the control group (Figure 3). These within-group changes are reported to describe the direction of the significant interaction effects rather than as independent evidence of intervention effectiveness.

**Figure 2:**
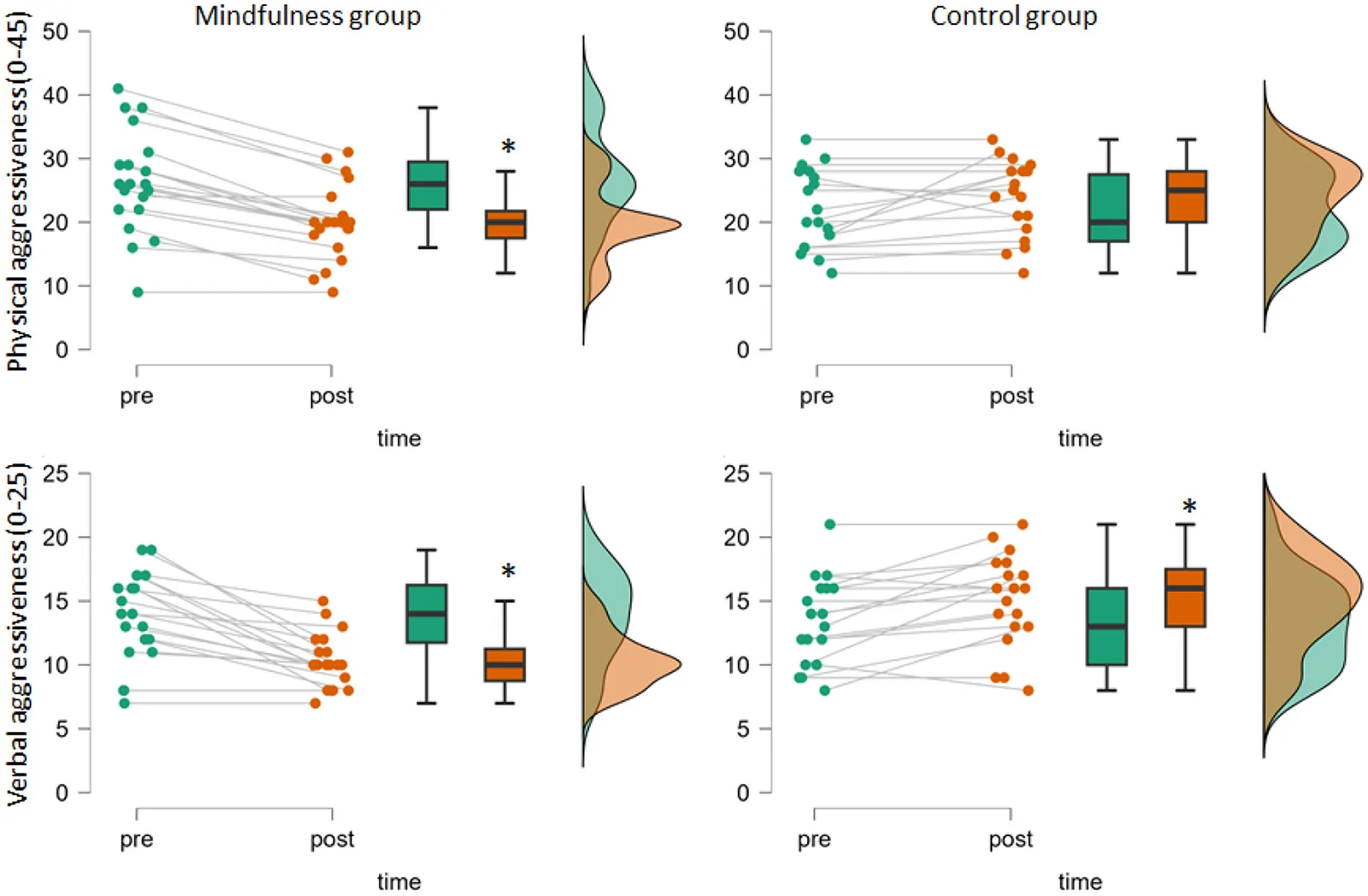
Individual responses on physical and verbal aggressiveness scores for both groups at pre and posttest. together with a box-and-whisker plot representing median, quartiles, and range per group and a density estimate.* indicates a significant difference from pre to posttest for this group.

**Figure 3:**
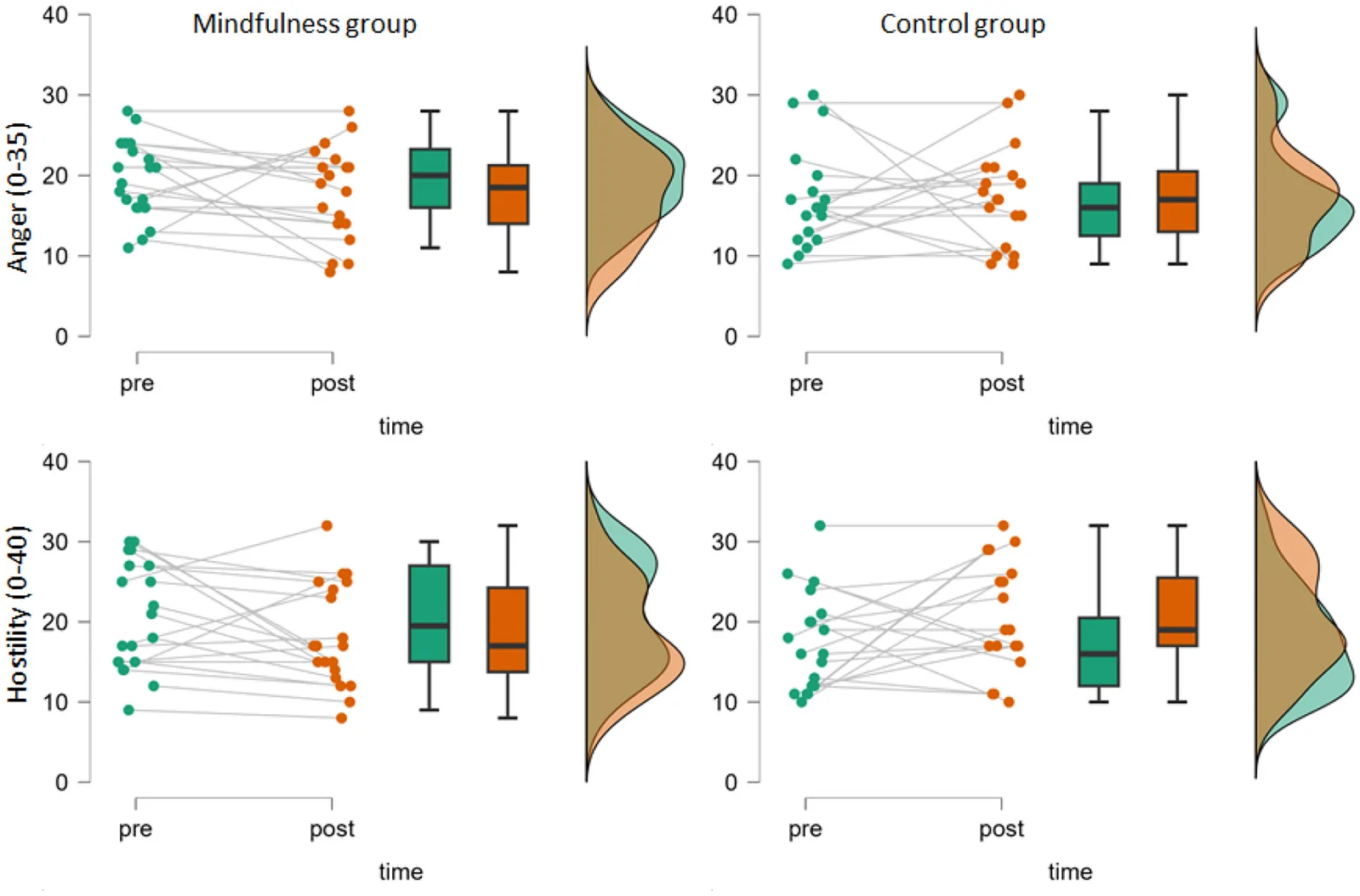
Individual responses on hostility and anger scores for both groups at pre and posttest together with a box-and-whisker plot representing median, quartiles, and range per group and a density estimate.

### 3.2 Barratt impulsivity scale

A significant effect of test occasion was found for motor impulsiveness (*F*_(1, 37)_ = 8.02, *p* = 0.007, $\eta_{p}^{2}$ = 0.18) and total impulsivity scores (*F*_(1, 37)_ = 5.79, *p* = 0.021, $\eta_{p}^{2}$ = 0.14), while significant group-by-time interaction effects were found only for non-planned impulsiveness (*F*_(1, 37)_ = 9.86, *p* = 0.003, $\eta_{p}^{2}$ = 0.21) and total impulsivity (*F*_(1, 37)_ = 5.61, *p* = 0.023, $\eta_{p}^{2}$ = 0.13). No significant group-by-time interaction was observed for motor impulsivity. No other significant effects were found for any of the parameters (*F*_(1, 37)_ ≤ 2.35, *p* ≥ 0.134, $\eta_{p}^{2}$ ≤ 0.06). Post hoc within-group comparisons revealed that the mindfulness group significantly decreased non-planned (−13.1%, Cohen's *d* = 0.80), motor (−11.6%, Cohen's *d* = 0.67) and total impulsivity scores (−10.5%, Cohen's *d* = 0.80), while the control group did not change the scores significantly (Figures 4, 5). Thus, although motor impulsivity decreased significantly within the mindfulness group, this change did not represent a significant differential effect relative to the control group (Cohen's *d* = 0.16, *p* = 0.55).

**Figure 4:**
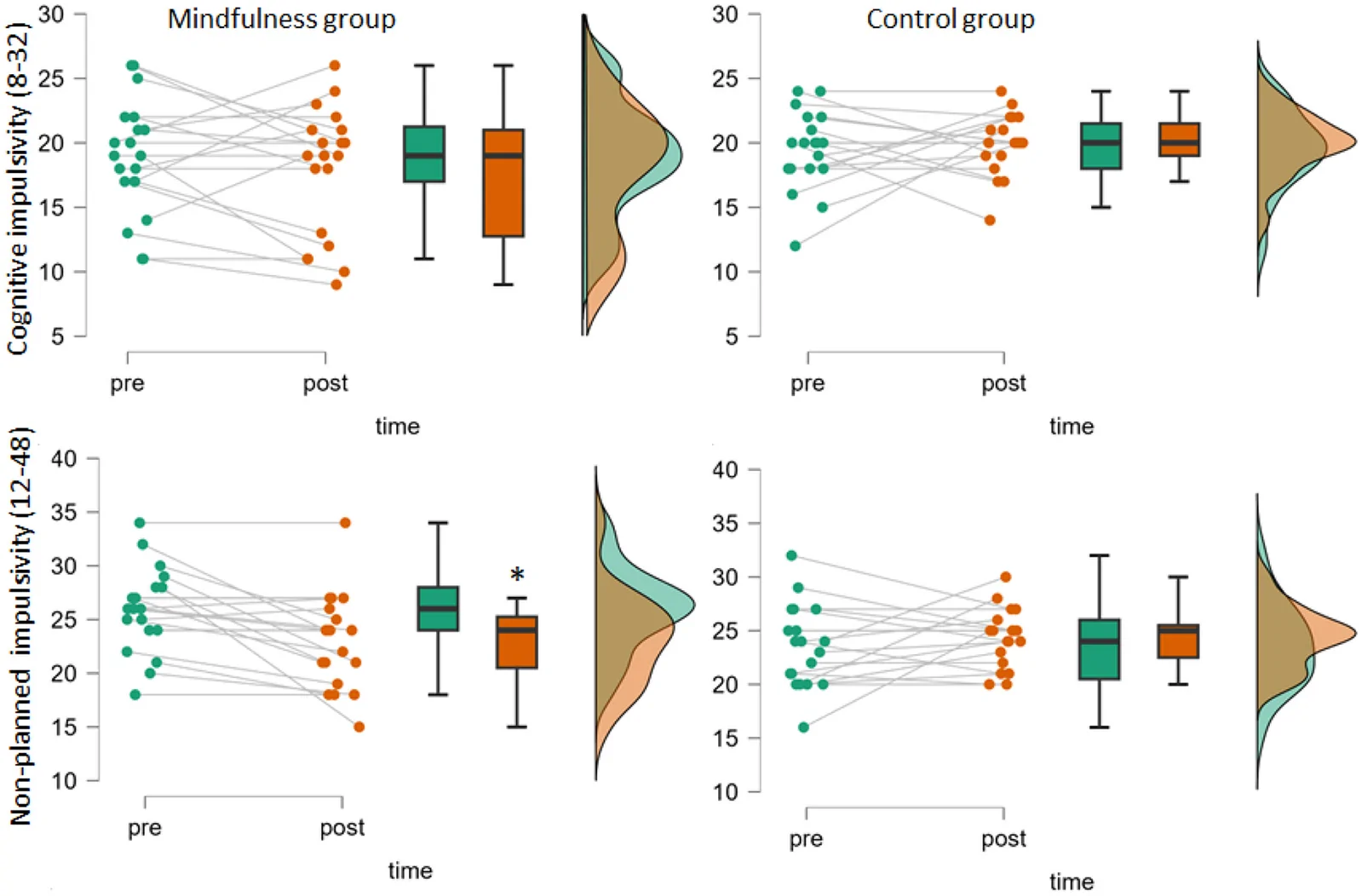
Individual responses on cognitive and non-planned impulsivity scores for both groups at pre and posttest together with a box-and-whisker plot representing median, quartiles, and range per group and a density estimate.* indicates a significant difference from pre to posttest for this group.

**Figure 5:**
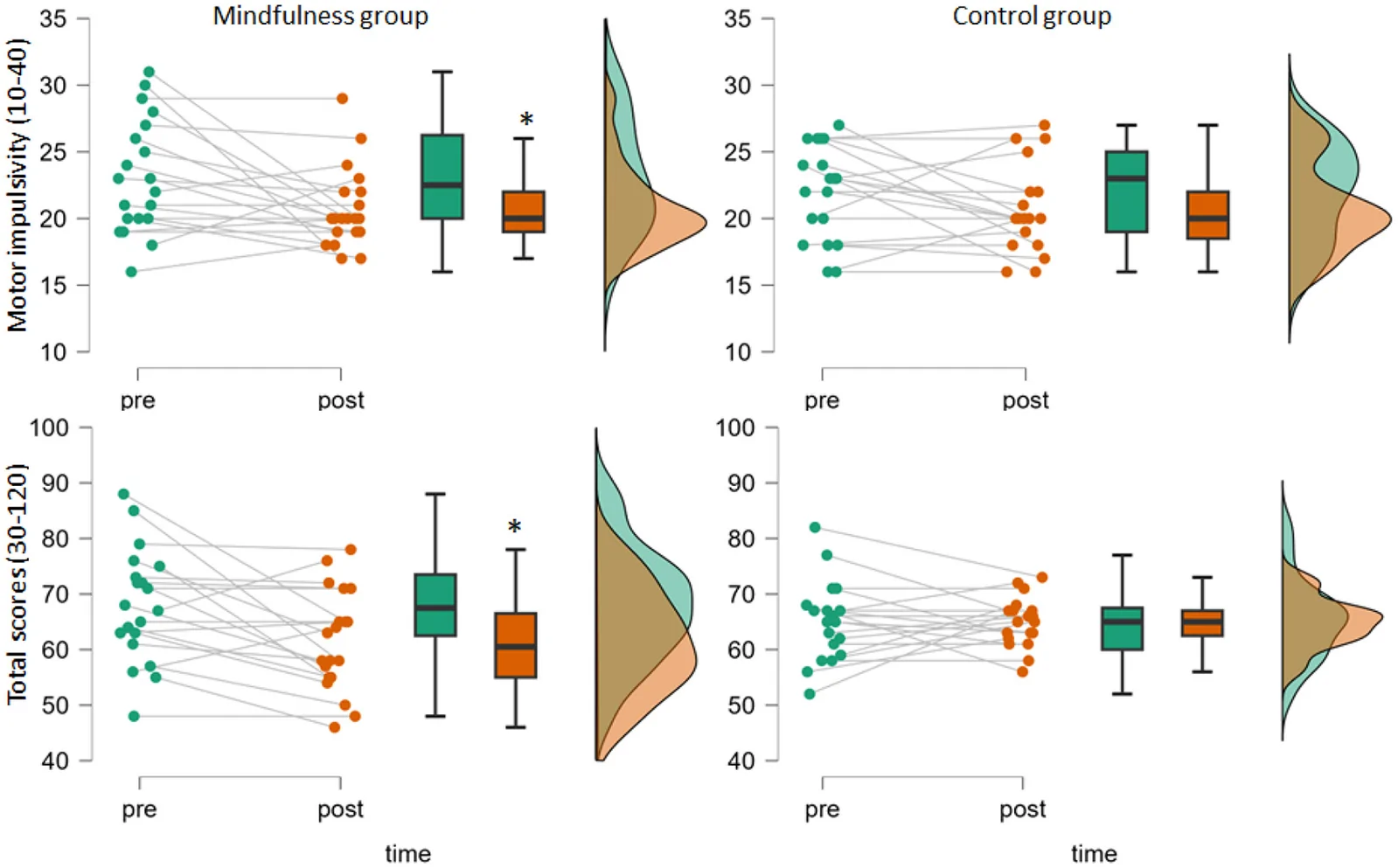
Individual responses on motor and total impulsivity scores for both groups at pre and posttest. together with a box-and-whisker plot representing median, quartiles, and range per group and a density estimate.* indicates a significant difference from pre to posttest for this group.

## 4 Discussion

The present study examined the effects of an 8-week mindfulness-informed psychoeducational intervention delivered during physical education sessions on general self-reported aggression and impulsivity among high school students. Results showed significant group-by-time interaction effects for verbal aggressiveness, physical aggressiveness, and hostility, indicating that changes over time differed between the intervention and control groups for these outcomes. These interactions were characterized by reductions in verbal aggressiveness (−33.5%) and physical aggressiveness (−32.4%) in the intervention group, as well as a decrease in hostility, whereas the control group showed increases in verbal aggressiveness and hostility over time. Regarding impulsivity, significant group-by-time interactions were observed for non-planned and total impulsivity, but not for motor impulsivity. Within the mindfulness group, significant pre-to-post reductions were observed in non-planned (−13.1%), motor (−11.6%), and total impulsivity (−10.5%) scores, while no significant pre-to-post changes were found in the control group. Thus, the reduction in motor impulsivity should be interpreted as a within-group change rather than evidence of a differential intervention effect relative to the control group. Overall, these findings support previous research demonstrating the potential benefits of mindfulness-based interventions in reducing maladaptive emotional and behavioral responses in adolescents (Franco et al., 2016; Hamidi et al., 2020; O'dean et al., 2025).

The reductions observed in physical and verbal aggression within the context of the significant group-by-time interactions may reflect improved behavioral and emotional regulation associated with the multicomponent intervention. Physical education environments frequently involve competition, performance pressure, and direct interpersonal interactions that can provoke frustration and aggressive responses (Burgueño et al., 2023; Lavega-Burgues et al., 2023). However, because aggression was assessed as a general self-reported disposition rather than specifically during PE lessons, these findings should not be interpreted as direct evidence of reduced aggressive behavior during PE activities.

Furthermore, a significant interaction effect for hostility was characterized by decreased hostility scores in the mindfulness group and increased hostility scores in the control group over time. This finding is consistent with previous research suggesting that mindfulness-based practices promote emotional stability and reduce emotional reactivity by encouraging present-moment awareness and non-judgmental acceptance of thoughts and emotions (Kemeny et al., 2012; Zhang and Zhang, 2023). Nevertheless, the present findings concern general self-reported hostility and do not establish that hostility specifically during PE activities was reduced. The findings therefore suggest that a mindfulness-informed psychoeducational intervention delivered within PE may be associated with differential changes in some dimensions of general aggression and impulsivity. However, because the intervention was multicomponent, the relative contribution of mindfulness-oriented vs. other psychological components cannot be determined. Regarding impulsivity, significant group-by-time interaction effects were observed for non-planned and total impulsivity, indicating differential changes between the intervention and control groups over time. Motor impulsivity also decreased significantly from pre- to post-test within the intervention group; however, because the group-by-time interaction for motor impulsivity was not significant, this within-group reduction cannot be interpreted as evidence that the intervention produced a greater change than the control condition. These findings are consistent with previous research indicating that mindfulness may be associated with executive functioning, attentional control, and inhibitory processes (Müller et al., 2021; Ge et al., 2025). Improved attentional regulation may help adolescents pause before reacting impulsively, but attentional control and related mechanisms were not directly measured in the present study and therefore cannot be established as mediators of the observed changes. In addition, the present study did not assess impulsive behavior specifically during PE tasks or peer interactions.

The significant interaction effects observed for non-planned and total impulsivity suggest that the mindfulness and control groups showed different patterns of change over time for these outcomes. In contrast, no significant group-by-time interaction effects were found for motor or cognitive impulsivity. Although motor impulsivity decreased significantly within the mindfulness group, this finding should be interpreted cautiously in the absence of a significant interaction effect. Cognitive impulsivity also showed no significant interaction effect, suggesting that the intervention did not produce a statistically detectable differential change in this outcome compared with the control condition. Further research with larger samples is needed to determine whether different dimensions of impulsivity respond differently to mindfulness-informed psychoeducational interventions. The 100% attendance rate and completion of all 16 planned sessions indicate high adherence to the intervention; however, feasibility and safety should be interpreted cautiously because participant engagement beyond attendance and potential adverse psychological reactions were not systematically assessed. From a practical perspective, integrating brief mindfulness-informed psychoeducational activities into physical education curricula may provide a feasible approach for supporting general emotional and behavioral self-regulation among adolescents. However, because the present outcomes were based on general self-reported dispositions, conclusions regarding reduced disruptive behaviors or improved social interactions specifically during PE require further investigation using PE-specific or observational measures.

Several limitations should be acknowledged. First, the relatively small sample size and the absence of an a priori sample-size calculation may have limited the statistical power to detect small group × time interaction effects and may also limit the precision and generalizability of the observed effects. In addition, the number of outcomes and statistical comparisons increases the possibility of false-positive findings despite the use of Holm-Bonferroni correction for *post-hoc* comparisons; therefore, the findings should be interpreted cautiously and require confirmation in adequately powered studies.

Second, the study relied primarily on self-reported psychological measures, which may be influenced by social desirability or response bias. No behavioral, teacher-rated, disciplinary, or direct observational measures of aggression or impulsivity were collected to corroborate the self-reported outcomes. Moreover, the internal consistency of some questionnaire subscales was relatively low, which may have increased measurement error and reduced the precision of the estimated effects. An additional limitation concerns the contextual interpretation of these outcomes. The AQ and BIS-11 were administered using their standard instructions and assess general self-reported aggression and impulsivity rather than behaviors specific to physical education. Participants were not instructed to respond specifically with reference to PE lessons. Therefore, although the mindfulness-informed psychoeducational intervention was delivered within PE classes, the observed changes should not be interpreted as direct evidence of reduced aggression or impulsivity specifically during PE. Future studies should incorporate PE-specific or observational behavioral measures to determine whether these effects extend to behaviors occurring within PE lessons.

Third, participants were recruited from a single school, limiting the applicability of the results to broader adolescent populations. Additionally, because participants from both groups belonged to the same class and interacted regularly throughout the intervention, potential contamination between groups cannot be excluded, as the transfer or practice of mindfulness-related techniques was not formally monitored. Participants and the facilitator could not be blinded to group allocation, and the facilitator was aware of the study hypotheses. Consequently, expectancy effects, Hawthorne effects, and other non-specific effects related to awareness of receiving the intervention cannot be excluded. Furthermore, the study used a passive rather than an active attention-matched control condition. Thus, facilitator attention, novelty, expectancy, and group engagement cannot be separated from the specific effects of the intervention components. Because the same facilitator was involved in intervention delivery, facilitator- or teacher-related effects may also have contributed to the observed findings. Future studies should use active attention-matched control conditions and, where feasible, separate intervention delivery from outcome assessment.

An additional limitation is the multicomponent nature of the intervention, which combined mindfulness-oriented practices (e.g., focused attention, conscious breathing, and body awareness) with positive affirmations, self-encouragement, performance visualization, and sport-success imagery. Consequently, the observed effects cannot be attributed specifically to mindfulness or to any individual component of the intervention. Furthermore, mindfulness and emotional regulation were not directly measured as manipulation checks or potential mediators. Therefore, the proposed roles of increased mindfulness, attentional control, and emotional regulation remain theoretical rather than empirically demonstrated in the present study. Future studies should include validated measures of mindfulness and emotional regulation and consider component-specific or dismantling designs to identify the relative contribution of the different intervention elements. Intervention fidelity was also not independently or quantitatively assessed using session checklists, observations, recordings, or independent ratings, which limits conclusions regarding consistency of intervention delivery despite the use of standardized content and the same facilitator.

Finally, the absence of long-term follow-up prevents conclusions regarding the persistence of the benefits observed over time. Future studies should include larger and more diverse samples across multiple schools and incorporate behavioral observations and physiological indicators of stress regulation, such as cortisol levels or heart rate variability. Future trials should also consider cluster randomization or recruiting intervention and control participants from separate classes to minimize the risk of contamination between groups. Longitudinal investigations are also needed to determine whether the observed changes in aggression and impulsivity can be maintained over extended periods and whether these effects translate into improved academic, social, and athletic outcomes.

## 5 Conclusion

Preliminary evidence from this small, single-school study suggests that an eight-week mindfulness-informed psychoeducational intervention delivered during physical education was associated with differential changes in selected self-reported dimensions of aggression and impulsivity, including verbal aggression, physical aggression, hostility, non-planned impulsivity, and total impulsivity, relative to the control condition. Although motor impulsivity decreased within the intervention group, the absence of a significant group-by-time interaction indicates that this finding should not be interpreted as evidence of a differential intervention effect.

These findings suggest that brief mindfulness-informed psychoeducational activities may have potential for integration within school physical education; however, recommendations for routine curricular implementation would be premature. The findings should be interpreted cautiously given the small sample size, single-class and single-school setting, reliance on self-reported outcomes, multicomponent nature of the intervention, and use of a passive control condition. Replication in adequately powered, multisite trials incorporating active attention-matched control conditions and behavioral outcome measures is required before stronger conclusions regarding effectiveness or routine implementation can be made.

## Data Availability

The raw data supporting the conclusions of this article will be made available by the authors, without undue reservation.
