# Supplementary material for: Effects of an eight-week mindfulness-informed psychoeducational intervention delivered during physical education lessons on self-reported aggression and impulsivity in adolescents
Source: Front Psychol. 2026 Oct 12;17:1958309. doi: 10.3389/fpsyg.2026.1958309 (PMC13696323; doi:10.3389/fpsyg.2026.1958309)
Supplement: Supplementary file 1 [file Table_1.docx]

|  | Mindfulness | | | Control | | | Time | | | interaction | | | group | | |
| --- | --- | --- | --- | --- | --- | --- | --- | --- | --- | --- | --- | --- | --- | --- | --- |
|  | pre | post | Δ | pre | Post | Δ | F | p | Eta | F | p | Eta | F | P | Eta |
| *Aggression* |  |  |  |  |  |  |  |  |  |  |  |  |  |  |  |
| Physical | 26.4±3.5 | 19.9±2.6 | -6.5±1.4 | 21.9±2.8 | 24.0±2.7 | 2.1±2.0 | 12.86 | < .001* | 0.258 | 48.1 | < .001* | 0.565 | 0.01 | 0.92 | <0.01 |
| Verbal | 13.8±1.6 | 10.3±0.9 | -3.5±1.2 | 13.2±1.6 | 15.0±1.7 | 1.8±1.1 | 3.766 | 0.06* | 0.092 | 40.798 | < .001* | 0.524 | 4.562 | 0.039* | 0.11 |
| Anger | 19.5±2.1 | 17.7±2.5 | -1.8±2.9 | 17.5±2.8 | 20.5±2.8 | 3.0±3.9 | 0.456 | 0.504 | 0.012 | 0.729 | 0.399 | 0.019 | 0.893 | 0.351 | 0.024 |
| Hostility | 20.6±3.0 | 18.2±2.8 | -2.4±3.0 | 17.2±2.8 | 17.4±3.1 | 0.2±3.7 | 0.069 | 0.794 | 0.002 | 4.681 | 0.037* | 0.112 | 0.056 | 0.814 | 0.002 |
| *Impulsivity* |  |  |  |  |  |  |  |  |  |  |  |  |  |  |  |
| Motor | 23.1±1.9 | 20.7±1.3 | -2.4±1.9 | 22.0±1.6 | 20.7±1.5 | -1.3±1.7 | 8.017 | 0.007* | 0.178 | 0.683 | 0.414 | 0.018 | 0.356 | 0.554 | 0.01 |
| Non planned | 26.0±1.7 | 23.0±1.9 | -3.0±1.6 | 23.5±1.7 | 24.3±1.2 | 0.8±1.7 | 3.354 | 0.075 | 0.083 | 9.858 | 0.003* | 0.21 | 0.26 | 0.613 | 0.007 |
| Cognitive | 18.9±1.9 | 17.8±2.2 | -1.1±1.8 | 19.5±1.4 | 19.9±1.1 | 0.5±1.8 | 0.548 | 0.464 | 0.015 | 0.448 | 0.507 | 0.012 | 2.353 | 0.134 | 0.06 |
| Total score | 67.9±4.4 | 61.5±4.0 | -6.5±4.2 | 65.0±3.2 | 64.9±2.0 | -0.1±3.1 | 5.792 | 0.021* | 0.135 | 5.606 | 0.023* | 0.132 | 0.018 | 0.893 | <0.01 |

**Supplementary Table.** Mean±95% CI of pre and post test per group with statistical analysis.
